# Adjuvant Therapy for High-Risk Stage II Melanoma: Current Paradigms in Management and Future Directions

**DOI:** 10.3390/cancers16152690

**Published:** 2024-07-29

**Authors:** Gracia Maria Vargas, Mohammad Saad Farooq, Giorgos C. Karakousis

**Affiliations:** Division of Endocrine and Oncologic Surgery, Department of Surgery, Perelman School of Medicine, University of Pennsylvania, Philadelphia, PA 19104, USA

**Keywords:** high-risk stage II melanoma, adjuvant therapy, pembrolizumab, nivolumab, immune checkpoint inhibitors, sentinel lymph node biopsy, recurrence-free survival, PD-1 inhibitors, targeted therapy, randomized clinical trials

## Abstract

**Simple Summary:**

Patients with stage IIB and IIC melanoma are considered to have high-risk localized disease and, in some cases, demonstrate worse recurrence-free and overall survival than patients with stage IIIA and even IIIB disease. The mainstay of treatment for patients with high-risk clinical stage II melanoma is surgical resection of the primary tumor and sentinel lymph node biopsy (SLNB), which confers accurate pathologic staging and prognostication for further treatment decisions. Building on the success of adjuvant therapy for stage III melanoma, the KEYNOTE-716 and CheckMate 76K trials found improved recurrence-free survival for patients with high-risk stage II melanoma with adjuvant pembrolizumab and nivolumab, respectively, after complete resection with SLNB. Given these findings, the National Comprehensive Cancer Network now recommends discussing adjuvant pembrolizumab or nivolumab with patients with high-risk stage IIB/C melanoma, weighing the risks of recurrence against the risks of treatment-related adverse events when making the decision regarding adjuvant therapy.

**Abstract:**

Melanoma is the fifth most common cancer in the United States and accounts for the majority of all skin cancer-related deaths, making it the most lethal cutaneous malignancy. Systemic adjuvant therapy for stage IIB-IV melanoma is now approved for patients who have undergone surgical resection, given the appreciable risk of recurrence and mortality in this patient population. Despite the lower stage, high-risk stage II melanoma (stage IIB/IIC) can often exhibit an even more aggressive course when compared to stage IIIA/IIIB disease, thus justifying consideration of adjuvant therapy in these patients. In this review, we highlight the current standard of practice for the treatment of stage IIB/C melanoma, with a focus on adjuvant therapies supported by published landmark clinical trials, including anti-PD-1 therapy. Notably, adjuvant therapies approved thus far in this patient population have demonstrated an improvement in recurrence-free survival, while their impact on overall survival is pending. Finally, this review highlights currently ongoing trials and future directions for research and treatment possibilities for high-risk clinical stage II melanoma.

## 1. Introduction

Cutaneous melanoma is the fifth most common cancer in the United States, comprising 5% of all new cancer diagnoses per year [1]. Melanoma accounts for approximately 65% of all skin cancer deaths, making it the most aggressive cutaneous malignancy [2]. The incidence of melanoma is increasing nationally and globally, particularly in individuals 65 years of age and older, who account for 54.4% of all new cases per year and 69.8% of all cutaneous melanoma deaths in the United States [3,4]. The lifetime risk of developing cutaneous melanoma is 1 in 53 for men and 1 in 34 for women; in aggregate, statistics estimate more than 8000 yearly deaths attributed to cutaneous melanoma in the USA alone [1,5]. Melanoma, therefore, imparts a significant and growing burden of disease on our society and healthcare system.

Melanoma prognosis varies widely according to cancer stage as well as other patient-level risk factors. Cutaneous melanoma staging follows the TNM classification as described by the American Joint Committee on Cancer (AJCC, 8th edition), ranging from stage IA (T1a) to stage IV (metastatic) [6]. AJCC criteria define high-risk stage II disease (IIB or IIC) as patients without nodal metastases but with high-risk tumor features. Specifically, stage IIB melanoma includes lesions that are T3b (Breslow depth 2–4 mm with ulceration) or T4a (>4 mm without ulceration). Stage IIC melanoma is defined by T4b lesions (>4 mm with the presence of ulceration). Patients with localized primary melanomas <1.0 mm in Breslow depth have 5-year melanoma-specific survival (MSS) rates upwards of 90%. By comparison, patients with stage IIB melanoma have 5- and 10-year MSS rates of 87% and 82%, respectively, while stage IIC melanoma has 5 and 10-year MSS rates of 82% and 75%, respectively [6]. Despite the absence of nodal or lymphatic spread, stage IIB/IIC melanoma can be characterized by a more aggressive clinical course and worse disease-specific survival than even stage IIIA disease (5- and 10-year MSS rates 93% and 88%, respectively) and in some cases stage IIIB disease (5- and 10-year MSS rates of 83% and 77%, respectively), highlighting the importance of identifying adjuvant treatments specifically for high-risk stage II melanoma [6,7,8].

While surgical resection remains the mainstay of treatment for localized melanoma, modern therapeutics with demonstrated efficacy in the metastatic setting, including immune checkpoint inhibitors and targeted therapy (BRAF and MEK inhibitors), have quickly made their way to the adjuvant setting for high-risk melanoma. Initially used primarily for stage III disease, a subset of these therapies are now approved in the high-risk stage II setting, and others are being further investigated, recognizing that many of these patients have a higher risk of relapse [6,7,8].

In this review article, we summarize the current treatment paradigms for high-risk stage II melanoma, including surgical management and surveillance strategies. We subsequently discuss the key trials and outcomes of adjuvant therapy for high-risk stage II melanoma in comparison with the more-studied outcomes in higher-stage melanoma and review ongoing studies and potential challenges with and opportunities for future systemic treatment options in patients with stage IIB/IIC melanoma.

## 2. Materials and Methods

A systematic search was conducted on the PubMed database for publications in peer-reviewed journals using the following keywords: “stage II” AND “melanoma” AND “adjuvant”, supplemented by citation mining. An emphasis was placed on systematic reviews, prospective studies, and randomized trials from 2018 to 2024.

## 3. Results

**Figure 1:**
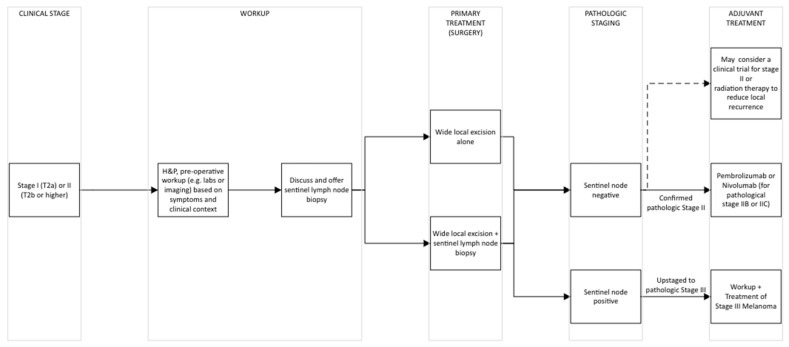
Treatment Algorithm for Stage IIB/IIC Melanoma. Adapted from NCCN Guidelines Version 2.2024. Melanoma: Cutaneous [9].

The National Comprehensive Cancer Network (NCCN) publishes frequently updated guidelines for the diagnosis and management of cutaneous melanoma, which are used internationally as the standard of care. Current management of clinical stage IIB/IIC melanoma consists of surgical management of the primary lesion via wide excision with sentinel lymph node biopsy (SLNB) and subsequent consideration of systemic adjuvant therapy and/or radiation therapy to the primary tumor site (Figure 1).

### 3.1. Surgical Management of High-Risk Stage II Melanoma

#### 3.1.1. Wide Local Excision

Surgery is the primary treatment for locally invasive melanoma, including high-risk stage II melanoma. Surgical resection of a primary cutaneous melanoma should be performed using the principles of wide excision, ensuring full-thickness excision to the level of the fascia and adequate peripheral surgical margins, according to the NCCN Guidelines [9]. Peripheral surgical margins for invasive melanoma are based on tumor thickness. For high-risk stage II (stage IIB/IIC) melanomas, which by definition have tumor thickness > 2.0 mm, peripheral surgical margins of 2 cm are recommended when feasible [10,11,12,13,14,15]. There is currently a phase III multi-center randomized controlled trial (MelMarT-II, NCT03860883), which is seeking to evaluate the adequacy of 1-cm margins in comparison to the current recommendation for 2-cm margins, with a primary outcome of disease-free survival for lesions > 2 mm in depth or 1–2 mm Breslow depth with ulceration (pT2b-pT4b) [16].

#### 3.1.2. Sentinel Lymph Node Biopsy

In addition to wide local excision of the primary lesion, a sentinel lymph node biopsy (SLNB) should be considered for patients with stage II melanoma. These patients generally have a risk of sentinel lymph node metastasis which exceeds 10%—far greater than the 5% clinical threshold for which SLNB is recommended. SLNB entails the selective removal of the lymph node(s) that receive direct drainage from the site of a primary melanoma, identified using techniques including lymphoscintigraphy with radiotracer (technetium 99m-labeled sulfur colloid) and intraoperative blue dye (methylene blue or isosulfan blue) for identification. Preoperative cross-sectional imaging is not routinely required for these patients, as it is unlikely to change surgical management in the absence of physical exam findings demonstrating evidence of metastatic disease [17]. However, baseline cross-sectional imaging may be useful for patients who may be considered for adjuvant systemic therapy and be recommended for future surveillance imaging.

The decision to proceed with SLNB should incorporate a consideration of the risks and benefits of the procedure specific to the patient, including the factors related to the patient’s clinical status as well as tumor pathology. For example, higher mitotic index, tumor thickness, and presence of lymphovascular invasion have been associated with an increased risk of SLN positivity, while increasing age has been associated with a lower probability of sentinel lymph node positivity [18,19]. Memorial Sloan Kettering Cancer Center and the Melanoma Institute Australia have both independently created risk calculators using factors such as age, Breslow thickness, mitotic rate, ulceration, histologic subtype, and lymphovascular invasion to assist clinicians in determining individual patients’ risk of positive SLNB [20,21]. Beyond taking into account these individual clinical and tumor-related risk factors for metastasis, it is important to consider additional factors that may impact the risk/benefit calculus of performing the procedure, including the risk of general anesthesia, patient frailty, and willingness of the patient to undergo adjuvant treatment regimens or surveillance regimens based on the outcomes of the SLNB procedure.

In the context of approved adjuvant immunotherapy for pathologic stage IIB/C disease, the utility of SLNB has been called into question for T3b and higher lesions, and efforts are ongoing to identify alternative forms of risk-stratification of these patients [22]. To date, however, the sentinel lymph node biopsy appears to continue to confer benefits for patients with stage IIB/IIC melanoma, namely: (1) confirming accurate staging based on pathologic assessment of regional nodal basins [23], (2) improving disease-free survival by removing micro-metastatic disease to improve regional nodal control [24,25], and (3) obtaining prognostic data to inform future treatment decisions, including adjuvant therapy and subsequent surveillance [26,27,28,29,30].

### 3.2. Adjuvant Therapy for High-Risk Melanoma

The introduction of immune checkpoint inhibitors and targeted therapy to the therapeutic landscape of advanced metastatic melanoma led to a rapid paradigm shift away from a longstanding focus on largely ineffectual chemotherapy-based regimens and poorly tolerated interferon-based regimens [31,32]. The rapid transition away from these treatments to markedly more effective treatments for unresectable melanoma brought a corresponding push to improve the management of surgically resected disease [33]. Following the successful implementation of immune checkpoint blockade to improve overall survival in unresectable melanoma, these drugs began to be applied in the adjuvant setting for completely resected stage III melanoma. This is particularly salient for patients with stage IIB/IIC melanoma, whose 5-year melanoma-specific survival is often worse than that of many patients with stage IIIA/IIIB melanoma [34].

#### 3.2.1. Development of Modern Adjuvant Therapy for Melanoma in Stage III Disease

Bolstered by the observation that melanoma is an immune-responsive tumor, immune checkpoint blockade was developed to harness immune function for the treatment of cancer. Ipilimumab (Yervoy, Bristol-Myers Squibb Company), which functions by inhibiting the co-inhibitory receptor CTLA-4, was the first immune checkpoint inhibitor (ICI) found to extend survival in metastatic melanoma, leading to its FDA approval for the treatment of melanoma in 2011 [32,35]. This was followed by nivolumab (Opdivo, Bristol–Myers Squibb Company) and pembrolizumab (Keytruda, Merck), two PD-1 inhibitors that up-regulate T-cell activity and were also found to improve outcomes in metastatic melanoma; these were approved for the treatment of metastatic melanoma in 2014 [35,36,37]. The most recent ICI to be approved for adjuvant treatment of melanoma is relatimab, a LAG-3 inhibitor that acts upon a co-inhibitory T-cell receptor that suppresses T-cell activation.

Ipilimumab was the first immune checkpoint inhibitor approved for adjuvant treatment of melanoma. The EORTC 18071 trial demonstrated improved recurrence-free survival (RFS) for resected stage III melanoma with adjuvant ipilimumab compared to placebo (HR 0.75) but showed high toxicity, with 54% of patients experiencing grade 3 or 4 adverse events and 43% experiencing immune-related adverse events (irAEs) [38,39]. Similarly, the ECOG 1609 trial found that a lower dose of ipilimumab (3 mg/kg) improved overall survival (HR 0.78) and relapse-free survival (HR 0.85) compared to high-dose interferon alfa (HDI). In the same trial, a higher dose of ipilimumab (10 mg/kg) did not show significant survival benefits and was associated with increased toxicity. Both trials reported common irAEs, such as gastrointestinal, hepatic, and endocrine issues, leading ipilimumab to fall out of favor due to its high toxicity [40].

The PD-1 inhibitors pembrolizumab and nivolumab were the second class of immune checkpoint inhibitors to be approved for the adjuvant treatment of melanoma. The EORTC 1325 trial (Keynote-054 trial) evaluated pembrolizumab versus placebo in patients with resected high-risk stage III melanoma (Table 1). The trial found that pembrolizumab prolonged RFS in the overall population (HR 0.56) as well as in the subgroup of patients with PD-L1-positive tumors (HR 0.57) [41]. There was no significant difference in the benefit of pembrolizumab based on PD-L1 status, BRAF-V600E mutation status, or the American Joint Committee on Cancer 8th edition (AJCC-8) stage, though the AJCC-8 stage was noted to be strongly predictive of RFS [42]. The rate of grade 3 or 4 irAEs was low at 7.7% (compared to 0.6% in placebo). The CheckMate-238 trial evaluated PD-1 inhibitor nivolumab versus CTLA-4 inhibitor ipilimumab as adjuvant therapy in resected advanced melanoma (stage IIIB, IIIC, and IV). This trial found that nivolumab was associated with a significant improvement in RFS (HR 0.65). Grade 3 or 4 treatment-related adverse events occurred in 14.4% of patients in the nivolumab group compared to 45.9% of patients in the ipilimumab group [43].

The LAG-3 inhibitor relatimab is the most recent immune checkpoint inhibitor approved for the adjuvant treatment of melanoma. In the Relativity-047 trial, a combination therapy of relatimab plus nivolumab (Opdualag, Brystol-Myers Squibb Company) was evaluated compared to nivolumab alone (Table 1), and a significant improvement was found in progression-free survival (HR 0.75) with relatimab-nivolumab compared to nivolumab alone [44]. Grade 3 or 4 treatment-related adverse events occurred in 18.9% of patients in the combination therapy group, compared to 9.7% of patients in the nivolumab group alone.

Therapeutic advances in the treatment of advanced melanoma have not been limited to immune checkpoint inhibitors; targeted therapies have also been assessed for their efficacy in the adjuvant setting. Specifically, the BRAF inhibitor dabrafenib (Tafinlar, Novartis Pharmaceuticals Corp) and MEK inhibitor trametinib (Mekinist, Novartis Pharmaceuticals Corp), which were shown to be efficacious in patients with metastatic BRAF-mutant melanomas, were brought into the adjuvant setting. The COMBI-AD trial evaluated the combination of dabrafenib and trametinib versus placebo in patients with BRAF V600E-mutant resected stage III melanomas (Table 1). The trial found that the dabrafenib/trametinib combination significantly improved relapse-free survival (HR 0.47); the combination also improved overall survival (HR 0.57), though this outcome did not surpass the predetermined analysis boundary [45]. Serious adverse events occurred in 36% of patients (10% in placebo). Moreover, 26% of patients experienced an adverse event leading to permanent discontinuation of the trial drug (3% in placebo). In the AVAST-M trial, the VEGF inhibitor bevacizumab (Avastin, Genentech, Inc., San Francisco, CA, USA) was evaluated versus observation as an adjuvant therapy for primarily stage III melanomas. This study found that the use of bevacizumab resulted in an improvement in the disease-free interval (HR 0.83) but no significant difference in overall survival (HR 0.97) or distant metastasis-free survival (HR 0.88) [46]. This regimen is seldom used in clinical practice in the context of immune checkpoint inhibitors and targeted therapy.

In sum, several systemic treatments have been approved for the adjuvant treatment of stage III melanoma, including several immune checkpoint inhibitors and targeted therapies. The introduction of pembrolizumab and nivolumab as alternatives to ipilimumab provided an adjuvant treatment option with similar to improved RFS outcomes based on hazard ratios with significantly lower toxicity. Following the approval of adjuvant immune checkpoint blockade for stage III melanoma, long-term prospective overall survival data are still lacking; however, retrospective data in patients with stage IIIB-D disease suggest improved overall survival outcomes among those receiving post-operative immunotherapy [47]. These findings are clinically significant in practice and led to the investigation of adjuvant therapies for patients with high-risk stage II (stage IIB/IIC) melanoma.

**Table 1:** Phase III Randomized Controlled Trials for Adjuvant Therapy in Stage III Melanoma.

Trial	Year *	# in Experimental Arm (N)	Adjuvant Therapy	Control	Outcome **	
					End Point	Hazard Ratio (95% CI)
EORTC 18071 [38,39]	2015	476 (951)	Ipilimumab	Placebo	RFS	0.75 (0.63–0.88)
CheckMate-238 [48,49]	2017	453 (906)	Nivolumab	Ipilimumab	RFS	0.72 (0.60 to 0.86)
EORTC 1325/ Keynote-054 [50,51]	2018	514 (1019)	Pembrolizumab	Placebo	RFS	0.61 (0.51 to 0.72)
AVAST-M [46]	2018	660 (1320)	Bevacizumab	Observation	OS	0.97 (0.78 to 1.22)
					DFI	0.83 (0.70 to 0.98)
ECOG 1609 [40]	2020	523 (1051)	Ipilimumab 3 mg/kg	High-dose interferon alfa	OS	0.78 (0.61 to 0.99)
					RFS	0.85 (0.66 to 1.09)
		511 (989)	Ipilimumab 10 mg/kg	High-dose interferon alfa	OS	0.88 (0.69 to 1.12)
					RFS	0.84 (0.65 to 1.09)
COMBI-AD [45]	2020	438 (870)	Dabrafenib + Trametinib	Placebo	OS	0.57 (0.42 to 0.79)
					RFS	0.51 (0.42 to 0.61)
Relativity-047 [44]	2022	355 (714)	Relatimab + Nivolumab	Nivolumab	PFS	0.75 (0.62 to 0.92)

Abbreviations: Recurrence-Free Survival (RFS), Overall Survival (OS), Progression-Free Survival, Disease-Free Interval (DFI); * Year of initial publication for the first data interval, when applicable. ** Data reported in this table are from the most recently published interval for each study as of 1 June 2024.

#### 3.2.2. Current Adjuvant Therapy Regimens for High-Risk Stage II Melanoma

In spite of the various systemic treatments that have been recently studied and approved to treat unresectable melanoma and have been brought into the adjuvant setting for stage III and higher disease, the only class of drugs with completed phase III randomized controlled trials and FDA approval for the treatment of stage IIB/IIC melanoma are PD-1 inhibitors, specifically pembrolizumab and nivolumab [52,53]. As with prior studies for more advanced melanoma, recurrence-free survival has been used as a valid surrogate endpoint for overall survival in these randomized studies (Table 2) [54].

Pembrolizumab was the first systemic therapy approved by the FDA for adjuvant treatment of adult and pediatric (>12 years of age) patients with stage IIB or IIC melanoma following complete resection. This approval was made following the completion of the KEYNOTE-716 trial, a randomized, double-blind, placebo-controlled trial comparing RFS in patients who received 2 mg/kg (up to 200 mg) of intravenous pembrolizumab every 3 weeks for up to one year with those who received placebo (Table 2). This study found a significant difference in disease recurrence in the pembrolizumab group compared to placebo (15% and 24%, respectively) at 20.9 months post-treatment [54]. While median RFS was not reached at the first and second assessment time points, the first interim (at approximately 14 months) found a significantly lower rate of recurrence or death among patients in the pembrolizumab group compared to placebo (HR 0.65, 95% CI 0.46–0.92) [55]. At its final study interval, the KEYNOTE-716 trial found a statistically significant improvement in recurrence-free survival and distant metastasis-free survival, with an estimated 36-month RFS of 76.2% for pembrolizumab versus 63.4% for placebo in patients with stage IIB disease (HR 0.62, 95% CI 0.42 to 0.92), and RFS 80.9% for pembrolizumab versus 68.1% for placebo in stage IIC disease (HR 0.57, 95% CI 0.36 to 0.88) [56]. A subgroup analysis found that pembrolizumab was associated with a statistically significant improvement in RFS in all major patient subgroups reported, namely both men and women and patients <65 years old and ≥65 years old [56]. At the first interim analysis, 16% of the pembrolizumab group patients experienced a grade 3–4 treatment-related adverse event, in comparison to 4% of the placebo group. The most common treatment-related adverse events were grade 1–2 in severity, including pruritus, fatigue, diarrhea, rash, arthralgia, and hypothyroidism. No deaths occurred due to the study treatment. These data supported the approval of pembrolizumab by the FDA for the treatment of high-risk stage II melanoma on 3 December 2021 [52].

Approximately two years after the approval of pembrolizumab for adjuvant treatment of completely resected high-risk stage II melanoma, nivolumab became the second PD-1 inhibitor approved for the same indications following completion of the CheckMate 76K trial. This randomized, double-blind phase III trial used a 2:1 randomization design to compare the recurrence rates of patients receiving 480 mg nivolumab every 4 weeks for 12 months versus placebo (Table 2). At its first interim analysis, nivolumab already demonstrated a significant and clinically meaningful improvement in recurrence-free survival compared with placebo, with 89.0% RFS in the nivolumab group versus 79.4% in the placebo group (HR 0.42, 95% CI 0.30–0.59), corresponding with a 58% reduction in the risk of recurrence or death in patients who received nivolumab compared to placebo [57]. A subgroup analysis found that nivolumab was associated with a statistically significant improvement in recurrence-free survival for both men and women and for patients <65 years old and ≥65 years old. The safety profile of nivolumab was very similar to that of pembrolizumab: at first interim analysis, 10.3% of the nivolumab group patients experienced a grade 3–4 treatment-related adverse event, in comparison to 2.3% of the placebo group. The most common treatment-related adverse events were the same as those with pembrolizumab, i.e., fatigue, pruritus, diarrhea, rash, hypothyroidism, and arthralgia. This study had 1/526 patients experience treatment-related death in the nivolumab group. Ultimately, the outcomes of this trial led to the FDA approval of nivolumab as an alternative PD-1 inhibitor appropriate for adjuvant treatment of completely resected high-risk stage II melanoma on 13 October 2023 [52].

While the above PD-1 inhibitors are the only current systemic therapies with FDA approval for adjuvant treatment of high-risk stage II melanoma, there are ongoing efforts to identify other systemic agents, such as targeted therapies, as potential future adjuvant treatments in this patient population. The AVAST-M trial included patients with resected IIB/IIC melanoma, though they were in the minority (27% of the study population). This study found that adjuvant bevacizumab significantly improved disease-free interval (HR 0.85, 95% CI 0.74–0.99) but had no effect on overall survival (HR 0.97, *p* = 0.76) or distant metastasis-free survival (HR 0.83, *p* = 0.03) [46]. Currently, there is a phase III trial for the use of a combination of the BRAF/MEK inhibitors encorafenib and binimetinib for the adjuvant treatment of BRAF V600-mutant stage IIB/IIC melanomas after complete resection. There are additional ongoing phase III trials for adjuvant combination therapies for high-risk patients with melanoma across stages (from high-risk stage II to IV), which hold the promise of even more effective adjuvant regimens (Table 3).

The KEYVIBE-010 (NCT05665595) trial assesses stage IIB-IV patients undergoing treatment with pembrolizumab in combination with vibostolimab (MK-7684A, an anti-TIGIT antibody) against pembrolizumab alone [59]. KEYNOTE-942 (NCT03897881) is a phase II trial currently assessing the efficacy of personalized cancer vaccine mRNA-4157 (also known as V940) in combination with pembrolizumab vs. pembrolizumab alone in high-risk stage II-IV melanoma. Preliminary data from this trial has shown increased recurrence-free survival in the vaccine treatment group, with an 8-month RFS of 78.6% in the V940/pembrolizumab combination arm versus 62.2% in the pembrolizumab monotherapy arm (HR 0.561, 95% CI 0.309 to 1.017 [61]. A follow-up phase III trial (V940-001, NCT05933577) is currently underway [60]. Data from these novel studies have the potential to further change treatment paradigms among patients with high-risk stage II melanoma.

### 3.3. Recurrence after Adjuvant Therapy for High-Risk Stage II Melanoma

Melanoma recurrence after adjuvant treatment of high-risk stage II melanoma remains a concern because of the aggressive nature of stage IIB and IIC melanoma, including the high risk of metastasis. Given the relatively new approval of adjuvant PD-1 therapy for high-risk stage II melanoma, the landmark KEYNOTE-716 and CheckMate76K trials currently serve as the only large-cohort reference points for clinicians to use for prognostication of recurrence. At the time of final analysis (median follow-up of 39.4 months), the KEYNOTE-716 trial found a 24.0% rate of recurrence in the pembrolizumab arm, compared to a 35.6% rate of recurrence among the placebo arm in the intention to treat population [56]. At the same interval, this trial also found a 15.2% rate of distant metastasis in the pembrolizumab arm, compared with a 24.3% rate of distant metastasis in the placebo arm. Notably, regional recurrence rates were not that dissimilar between the two trial arms, 17.7% in the pembrolizumab arm versus 25.2% in the placebo arm. The CheckMate76K trial, at its data cutoff with a median of 15.8 months, found a 12.5% recurrence rate in the nivolumab arm, in comparison to a 26.1% rate of recurrence in the placebo arm. Distant metastasis occurred at a rate of 8.0% in the nivolumab arm, compared with 15.5% in the placebo arm; further, only 3.4% of patients treated with nivolumab had multiple lesions at the time of recurrence, compared to 9.1% of patients who were treated with placebo [57].

The treatment of melanoma recurrence in high-risk stage II patients previously treated with adjuvant therapies should take into consideration the clinicopathologic presentation of the recurrence as much as the disease’s prior behavior in response to adjuvant treatment. Recurrent melanoma in patients initially presenting stage IIB or IIC disease has been found to be more likely to recur in regional nodes, followed by lung and finally, in-transit locations [62]. Stage IIB and IIC melanomas have also been shown to have the highest probability of recurrence within the first three years of diagnosis [63]. Clinicians should take into consideration the location(s) and timing of recurrence when determining a subsequent treatment plan, which should be guided by the pattern of recurrence [9]. In the setting of prior systemic therapy, the choice of systemic therapy for treatment of the recurrence should take into consideration the timing and pattern of recurrence in relation to the adjuvant therapy. Patients who experienced disease progression during or shortly after completion of adjuvant therapy may benefit from a new adjuvant regimen from a different class. In contrast, those patients who experienced a level of disease control (i.e., complete response, partial response, or stable disease) without lasting treatment-related adverse effects and who experience recurrence or progression over 3 months after treatment discontinuation may be considered for resumption of the same agent or class of agents upon recurrence [9].

### 3.4. Surveillance and Follow-Up after Adjuvant Therapy for Stage II Melanoma

Patients with stage IIB/C melanoma should undergo frequent follow-ups with history and physical exams, including dermatologic skin examination, with consideration of imaging as an adjunct modality, to regularly assess the patient for recurrence or metastatic disease, as 27% of stage II melanoma patients recur within 5 years [63]. NCCN guidelines suggest a range of frequency for follow-up (every 3–6 months for 2 years, then every 3–12 months for 3 years, then annually), with the discretion afforded to the clinician based on case-specific risk factors [9]. While not strictly prescribed, routine imaging surveillance should be considered in patients who are at high risk of recurrence, as only 60% of recurrences in stage II patients are detected by the patient, while 27% are detected by imaging. Commonly, patients undergo biannual cross-sectional imaging with a CT scan of the chest, abdomen, and pelvis for surveillance. Occasionally, whole-body PET/CT may be used to identify disease in other anatomic locations or to characterize CT findings. Brain imaging with MRI is used selectively in patients with clinical signs or symptoms concerning for brain metastasis. While the ultrasound modality has been shown to be the most effective in detecting lymph node metastases, routine ultrasound surveillance of lymph node basins is reserved for patients with positive lymph nodes (i.e., pathologic stage III melanoma) [64,65].

In patients not receiving adjuvant therapy, which represents the majority of stage IIB/IIC patients currently, surveillance imaging may help in the detection of distant disease and may be particularly valuable in patients who have a lower rate of self-detection [63]. Clinicians may also take into consideration other tumor and patient factors when considering the frequency of surveillance imaging in high-risk stage II patients. It should be noted that the clinical value of early detection of metastatic or recurrent disease is not established [8], although some data suggest that earlier detection of metastatic disease may lead to improved outcomes [66].

Beyond routine imaging and skin tests, novel adjunctive methods have been proposed to assist clinicians with early detection of recurrence, such as circulating tumor DNA (ctDNA). One study assessing ctDNA in patients with high-risk stage II or III resected melanoma with BRAF or NRAS mutations enrolled in the AVAST-M trial found that detectable ctDNA was a significant predictor for decreased disease-free interval and distant metastasis-free interval, even after adjusting for performance status and disease stage [67].

## 4. Discussion

Significant progress has been made since the initial introduction of ICI and targeted therapies in the treatment of melanoma, with randomized trial data supporting the use of PD-1 inhibitors in high-risk stage II melanoma. Alongside the growing number of systemic agents for melanoma, the need to stratify and differentiate treatment modalities based on patient-specific clinicopathologic factors and toxicity/risk profile optimization has become more evident.

The KEYNOTE-716 and CheckMate-76K trials were the first to specifically assess the efficacy of adjuvant pembrolizumab and nivolumab, respectively, in high-risk stage II melanoma. Together, these trials now form the standard adjuvant treatment recommendation for high-risk stage II cutaneous melanoma, having demonstrated significant improvements in recurrence-free survival with PD-1 inhibition [9,54,55,56]. It is important to note, however, that most patients with stage IIB/IIC melanoma can be cured by surgical treatment alone. Thus, the decision for adjuvant treatment should be carefully weighed against the risk of adverse events. Given the lower risk of recurrence in this patient population compared to higher stage III/IV disease, the acceptable threshold for adverse events should also be lower in this cohort of patients, especially since toxicities from ICIs can lead to flare-ups, irreversible damage, and/or death, particularly in patients with pre-existing autoimmune conditions [68,69]. Given this, optimizing risk stratification for melanoma patients that may be suitable candidates for adjuvant treatment is still an ongoing avenue of investigation.

ICI therapy in melanoma has been associated with potentially significant acute and chronic immune-related adverse events (irAE), heightening the importance of judicious and precise implementation of these therapies [70]. Long-term irAEs and health-related quality of life outcomes (HRQoL) have been studied in patients undergoing anti-PD1/PD-L1 therapy for multiple cancers, including melanoma. In a single-center retrospective analysis of 135 melanoma patients who had undergone ICI therapy between 2009–2017, 71% of patients developed acute irAE during the treatment period, with the most common being dermatitis (23%), colitis (12.6%), arthritis (11.85%), hepatitis (10.37%), and hypophysitis (5.19%). The most common chronic irAEs in the melanoma group included hypothyroidism (9.45%), adrenal insufficiency (4.44%), and arthritis (4.44%). Patient-reported outcomes were overall favorable; however, younger age and the need for subsequent therapy were significantly associated with worse HRQoL metrics [71]. Acute and chronic cardiovascular sequelae of ICI therapy (e.g., myocarditis, pericarditis, and cardiomyopathy) are particularly dangerous, with myocarditis occurring in up to 1.14% of patients with mortality reaching 50%. A combination ICI therapy of anti-CTLA4 and anti-PD1/PD-L1 appears to increase the risk of cardiac complications [72]. Though typical onset is within 30 days of exposure to ICIs, more chronic symptoms have also been reported. In a single-center matched cohort analysis, patients with cancer receiving ICI therapy had a 3-fold increase in ischemic stroke, myocardial infarction, and coronary intervention; these findings were associated with increased aortic plaque volumes [73]. The aforementioned findings emphasize the importance of treatment stratification for melanoma patients.

Further investigation into the prediction of the toxicities and side effect profiles associated with ICI therapy specifically in stage IIB/C patients, will also be important, with grade 3–4 treatment-related events currently occurring in 10.3 to 16% of patients receiving anti-PD-1 therapy [55,57]. Importantly, however, absolute adverse event rates to anti-PD-1 therapy appear similar when comparing treatment in the adjuvant stage IIB/C setting versus in the adjuvant stage III/IV setting. The rates of irAEs for adjuvant pembrolizumab in high-risk stage II disease versus stage III/IV disease (KEYNOTE-006) were 16% and 13.3%, respectively [36,55]. Similarly, comparing nivolumab as treatment for high-risk stage II disease versus for stage III/IV disease (CheckMate-238), irAEs were also similar at 10.3% and 14.4%, respectively [37,57]. These data suggest that the side-effect profile for adjuvant treatment of high-risk stage II disease is similar relative to treatment of stage III/IV disease with the same agents.

Notably, data from trials looking at combination immunotherapy, specifically in stage IIB/IIC disease, are currently lacking. CheckMate 915 is a recent trial assessing combination therapy of nivolumab/ipilimumab vs. nivolumab alone in stage IIIB-IV melanoma. Interestingly, at a follow-up time of 24 months, there was no difference in recurrence-free survival between the two groups; however, combination therapy exhibited significantly increased adverse events [43]. Given these findings and the parallels in toxicity for monotherapy between adjuvant treatment of stage III/IV and stage IIB/IIC disease, combination adjuvant immunotherapy with anti-CTLA4 and anti-PD1 therapy may have an acceptable toxicity profile in the adjuvant space in the lower risk stage II melanoma setting. Combination therapies with more favorable toxicity profiles may represent an important area of further investigation.

While established data now exist for anti-PD-1-based immunotherapy in the treatment of high-risk stage II melanoma, data are still lacking on the value of targeted therapies (i.e., BRAF inhibitors and MEK inhibitors). The COMBI-AD trial has established the usage of dabrafenib/trametinib in the treatment of BRAF V600E mutant stage III melanoma [74,75]. As a corollary, the currently ongoing COLUMBUS-AD will assess a combination of encorafinib (BRAF inhibitor) and binimetinib (MEK inhibitor) in stage IIB/IIC melanoma [58]. Targeted BRAF/MEK inhibition has demonstrated lower rates of adverse events in comparison to immunotherapy and thus remains an exciting area of future investigation [74].

Though surgery and adjuvant therapy have become a cornerstone of advanced melanoma treatment, recent landmark studies in neoadjuvant therapy have shed light on its potential advantages, including a more robust/sustainable anti-tumor response, as well as downstaging of tumors and avoidance of surgical treatment completely. Various combinations/regimens of neoadjuvant therapies have been studied for stage III+ disease; however, no current data exist for stage IIB/IIC melanoma [76]. The recent SWOG 1801 and NADINA trials have both demonstrated the importance of neoadjuvant immunotherapy in the clinical stage III setting, with significant improvements demonstrated in event-free survival [77,78]. The NCT03757689 phase II trial is currently underway to assess sentinel lymph node positivity rates as the primary endpoint, as well as recurrence-free survival and safety/tolerability as secondary endpoints, in patients with clinical stage IIB/IIC melanoma undergoing PD-1 inhibition therapy [79].

Gene expression profile (GEP) testing is a relatively new technique that has been employed to identify stage I-III melanoma patients who are at higher risk for metastasis with promising results [80]. Several commercial GEP testing products have been developed and studied using predominantly retrospective data to assess their efficacy in risk stratification for different outcomes in patients with clinically localized melanoma, including the DecisionDx 31-GEP test, the SkylineDx Merlin CP-GEP assay, and the MelaGenix GEP score [81,82,83]. Data to date have demonstrated that these assays hold the potential for risk stratification when selecting patients for SLNB (i.e., those with low, or <5%, risk of SLN positivity) [84,85,86]. Prospective studies for validation and application of these assays are ongoing via various registry studies and trials, including the DECIDE study for prospective validation of the 31-GEP assay for selection of patients who may consider forgoing SLNB, the MERLIN-001 (NCT04759781) study for prospective validation of the CP-GEP test, and the phase III NivoMela trial (NCT04309409) which is using the MelaGenix GEP score to identify patients at high risk for recurrence following adjuvant nivolumab in stage IIA-IIC melanoma [84,87,88]. Current GEP profiling data still remain to be incorporated into prognostic and predictive paradigms in the care of melanoma patients, and additional prospective comparative analysis is needed to determine their clinical utility and better align risk–benefit-based treatment plans [89]. Given that the precise impact of GEP testing in clinical care has not yet been well defined, routine GEP testing has not yet been incorporated into major clinical guidelines for melanoma care, though the outcomes of ongoing prospective trials are eagerly anticipated.

Circulating tumor DNA (ctDNA) is another exciting avenue for investigation not only for post-treatment surveillance but also for the expeditious identification of disease relapse in potential candidates for additional adjuvant treatment in melanoma [90]. The DETECTION (NCT04901988) phase II/III trial is currently studying patients with stage IIB/IIC melanoma using ctDNA to see if earlier detection and treatment of recurrent disease with adjuvant nivolumab yields an overall survival benefit; initial results from this study are pending [91].

Newer investigation directions are also currently underway, with multiple phase III trials. The KEYVIBE-010 (NCT05665595) trial assesses stage IIB-IV patients undergoing treatment with pembrolizumab in combination with vibostolimab (MK-7684A, an anti-TIGIT antibody) against pembrolizumab alone [59]. KEYNOTE-942 (NCT03897881) is a phase II trial currently assessing the efficacy of personalized cancer vaccine mRNA-4157 (also known as V940) in combination with pembrolizumab vs. pembrolizumab alone in high-risk stage II-IV melanoma; preliminary data from this trial has shown increased recurrence-free survival in the vaccine treatment group [61]. A follow-up phase III trial (V940-001, NCT05933577) is currently underway [60]. Data from these novel studies have the potential to further change treatment paradigms among patients with stage II melanoma.

## 5. Conclusions

- Patients with stage IIB/IIC melanoma are at high risk for recurrence, frequently demonstrating worse melanoma-specific survival compared to stage IIIA and IIIB patients.
- Patients with high-risk clinical stage II melanoma benefit from sentinel lymph node biopsy for regional disease control, accurate pathologic staging, and prognostication and risk stratification to aid in adjuvant treatment decision-making.
- There is high level (category 1) evidence to support adjuvant treatment for improved recurrence-free survival in high-risk stage II melanoma with PD-1 inhibition (pembrolizumab or nivolumab) after wide local excision with sentinel lymph node biopsy.
- While the KEYNOTE-716 and CheckMate 76K studies both found improvements in recurrence-free survival for patients with stage IIB/IIC melanoma with a favorable risk profile for anti-PD1 therapy, the decision to give adjuvant therapy should be considered on an individual basis in this patient population.
- Further studies investigating alternate adjuvant regimens, including combination regimens, as well as neoadjuvant regimens for high-risk stage II melanoma, are ongoing. Accurate risk stratification will remain an important component in determining the appropriateness of systemic treatment escalation in this patient population in the future.

## Figures and Tables

**Table 2:** Phase III Randomized Controlled Trials for Adjuvant Therapy in Stage IIB/IIC Melanoma.

Trial	Year *	# in Experimental Arm (N)	Adjuvant Therapy	Control	Outcome **	
					End Point	Hazard Ratio (95% CI)
KEYNOTE-716 [55,56]	2022	487 (976)	Pembrolizumab	Placebo	RFS	0.65 (0.46 to 0.92)
CheckMate 76K [57]	2023	526 (790)	Nivolumab	Placebo	RFS	0.42 (0.30 to 0.59)

* Year of initial publication for the first data interval, when applicable. ** Data reported in this table are from the most recently published interval for each study as of 1 June 2024.

**Table 3:** Ongoing Phase III Trials for Adjuvant Therapy in Stage IIB/IIC Melanoma.

Trial	Adjuvant Therapy	Control	End Point	AJCC 8th Ed. Melanoma Stage
COLUMBUS-AD [58]	Encorafenib + Binimitenib	Placebo	RFS	IIB/IIC
KEYVIBE-010 [59]	Pembrolizumab + Vibostolimab	Pembrolizumab	RFS	IIB to IV
V940-001 [60]	Individualized neoantigen therapy mRNA-4157 (V940) + Pembrolizumab	Pembrolizumab	RFS	IIB to IV
